# Prediction of anticancer drug resistance using a 3D microfluidic bladder cancer model combined with convolutional neural network-based image analysis

**DOI:** 10.3389/fbioe.2023.1302983

**Published:** 2024-01-10

**Authors:** Sungho Tak, Gyeongjin Han, Sun-Hee Leem, Sang-Yeop Lee, Kyurim Paek, Jeong Ah Kim

**Affiliations:** ^1^ Research Center for Bioconvergence Analysis, Korea Basic Science Institute, Cheongju, Republic of Korea; ^2^ Graduate School of Analytical Science and Technology, Chungnam National University, Daejeon, Republic of Korea; ^3^ Department of Biomedical Sciences, Dong-A University, Busan, Republic of Korea; ^4^ Department of Health Sciences, The Graduate School of Dong-A University, Busan, Republic of Korea; ^5^ Center for Scientific Instrumentation, Korea Basic Science Institute, Daejeon, Republic of Korea; ^6^ Department of Bio-Analytical Science, University of Science and Technology, Daejeon, Republic of Korea; ^7^ Chung-Ang University Hospital, Chung-Ang University College of Medicine, Seoul, Republic of Korea

**Keywords:** bladder cancer, drug resistance, organ-on-a-chip, convolutional neural network, step decay learning rate

## Abstract

Bladder cancer is the most common urological malignancy worldwide, and its high recurrence rate leads to poor survival outcomes. The effect of anticancer drug treatment varies significantly depending on individual patients and the extent of drug resistance. In this study, we developed a validation system based on an organ-on-a-chip integrated with artificial intelligence technologies to predict resistance to anticancer drugs in bladder cancer. As a proof-of-concept, we utilized the gemcitabine-resistant bladder cancer cell line T24 with four distinct levels of drug resistance (parental, early, intermediate, and late). These cells were co-cultured with endothelial cells in a 3D microfluidic chip. A dataset comprising 2,674 cell images from the chips was analyzed using a convolutional neural network (CNN) to distinguish the extent of drug resistance among the four cell groups. The CNN achieved 95.2% accuracy upon employing data augmentation and a step decay learning rate with an initial value of 0.001. The average diagnostic sensitivity and specificity were 90.5% and 96.8%, respectively, and all area under the curve (AUC) values were over 0.988. Our proposed method demonstrated excellent performance in accurately identifying the extent of drug resistance, which can assist in the prediction of drug responses and in determining the appropriate treatment for bladder cancer patients.

## 1 Introduction

Bladder cancer (BC) is among the ten most common cancers worldwide and is classified into various stages, types, and grades according to tumor characteristics and the extent of invasion (Chen et al., 2019; Tran et al., 2021). The most common subtype of BC is non-muscle-invasive bladder cancer (NMIBC) found in the inner lining of the bladder (Shelley et al., 2012; Sylvester et al., 2021). NMIBC exhibits relatively high survival rate but tends to recur at high rates, and 10%–15% of cases progresses to invasive BC and metastasizes to other organs (Chen et al., 2019). Therefore, the choice of treatment for BC, depending on the type or stage, is crucial (Kamat et al., 2016; Tran et al., 2021). However, anticancer drug treatments have some limitations. First, the efficacy and response of drugs can significantly vary among individual patients, along with a wide range of diverse side effects. Another important challenge is the high frequency of resistance to anticancer drugs, leading to poor survival outcomes (Massari et al., 2015; Roh et al., 2018).

To achieve complete cancer treatment and suppress metastasis, it is crucial to identify individualized drug efficacy and progression patterns for each patient. In addition, it is necessary to develop a validation system for personalized anticancer drugs that can maximize treatment efficacy and minimize side effects by predicting the possibility of anticancer drug resistance.

Various next-generation technologies have recently been developed for this purpose. For example, the active development of validation systems based on organ/organoid-on-a-chip technology using patient-derived cells is in progress for drug efficacy assays (Kondo and Inoue, 2019; Ma et al., 2021; Lee et al., 2022; Driver and Mishra, 2023; Paek et al., 2023). These systems mimic the unique *in vivo* environment of individual patients, contribute to the efficient selection and evaluation of personalized anticancer drugs, and provide evidence for new diagnostic techniques or therapeutic drugs. Additionally, studies that rely on the patient’s genome have been conducted to identify gene expression patterns or mutations, enabling prognostic prediction for patient treatment (Li et al., 2015; Nagata et al., 2016; Tran et al., 2021). Furthermore, there has been a recent rapid evolution of artificial intelligence (AI) technologies that enable the efficient analysis of vast amounts of data (Parlato et al., 2017; Nguyen et al., 2018; Cascarano et al., 2021).

AI technologies have gained attention because they provide accurate and rapid analytical tools for overcoming the limitations of current cancer treatments. In BC, the diagnosis, evaluation of drug efficacy, and limitation of treatments have been investigated based on clinical tissues, liquid biopsy, and laboratory data combined with AI techniques (Borhani et al., 2022). AI is being used to predict BC using clinical laboratory data (Tsai et al., 2022) or support the diagnosis in cystoscopic images or computed tomography images of BC patients (Garapati et al., 2017; Ikeda et al., 2020). Machine learning studies can be used to evaluate for various clinical data to predict long-term outcomes, such as cancer recurrence and survival after radical cystectomy (Hasnain et al., 2019). Cellular analysis has been conducted using atomic force microscopy on cells collected from urine, and computer-supported machine learning data analyses have been employed (Sokolov et al., 2018).

In this study, we employed BC models on an organ-on-a-chip platform and applied deep learning to propose a new platform for determining the degree of anticancer drug resistance. In this study, we utilized the bladder cancer cell lines (Mun et al., 2022), developed to have four differential levels of resistance against gemcitabine (GEM), an active anticancer drug against BC, and these cells were cultured in a microfluidic chip-based 3D cell culture platform to recreate BC models with different morphological characteristics associated with their anticancer drug resistance. We employed convolutional neural network (CNN) models (Krizhevsky et al., 2012) and evaluated their performance. Using these models, we investigated how they effectively discriminated between different images obtained from our 3D cell culture platforms with different levels of drug resistance and determined the extent of discrimination achieved. When applied to clinical samples, this system can be utilized as a valuable validation system to provide criteria and facilitate better decision-making regarding optimal treatment strategies for patients.

## 2 Materials and methods

### 2.1 Fabrication of the microfluidic chip and 3D cell culture

Details regarding the design and soft lithographic fabrication process of the microfluidic chip for 3D BC cell culture can be found in our previous reports (Jeong et al., 2020; Hong et al., 2021). Briefly, the microfluidic chip consisted of top (polydimethylsiloxane [PDMS], 5–6 mm in thickness) and bottom (square cover glass, 24 mm × 24 mm) layers. The microfluidic channels constructed on the chips were filled with 1 mg mL^−1^ of poly-D-lysine hydrobromide (Sigma-Aldrich, United States) for 4 h for surface coating. Finally, the microfluidic channels were rinsed with distilled water and completely dried before use.

### 2.2 Cell culture and BC model using microfluidic chips

In our previous study, human BC cell lines with four levels of anticancer resistance were established (Mun et al., 2022). Briefly, after exposing parental T24 (American Type Culture Collection, United States) cells (P0) to GEM at an initial concentration of 1,500 nM, only surviving cells were re-cultured. During repetitive subcultures, GEM-resistant strains at each level were prepared until a total of 15 phases were reached. In this experiment, the GEM-resistant bladder cancer (GRC) cell lines, including the parental phase P0, early phase P3, intermediate phase P7, and late phase P15, were designated as levels 0, 1, 2, and 3, respectively. Using these cell lines, 3D cell culture and tumor migration tests were performed in the microfluidic chips; the details are described in our previous report (Mun et al., 2022). Briefly, the central channel of the chip was filled with type I collagen (2 mg mL^−1^, Corning, United States), and two media channels placed on both sides were coated (type I collagen solution, 35 μg mL^−1^ in PBS) to enhance the attachment of cells on the channel surface. A suspension of GRC cells (2 × 10^6^ cells mL^−1^) at each condition (P0, P3, P7, and P15) was introduced into one of the media channels and incubated for attachment. After 2 h, a suspension of HUVECs (2 × 10^6^ cells mL^−1^) was introduced into the other media channel on the opposite side of the GRC channel. The two cell types were co-cultured for 4 days in a chip with a 1:1 mixture of media from each cell.

### 2.3 Immunostaining and image analysis

To assess the migration of GRC cells cultured in microfluidic chips, the cells were fixed with 4% paraformaldehyde at room temperature for 20 min and permeabilized with 0.1% Triton X-100 for 20 min. Actin filaments and nuclei were stained with phalloidin-594 (1:40; Invitrogen, United States) and Hoechst 33342 (1:1500; Thermo Fisher Scientific, United States), respectively. Fluorescent cell images were obtained using a high-content screening microscope (Celena X; Logos Biosystems, Republic of Korea). A set of images acquired from the chips, representing cell mobility and the surrounding tissue, was taken at 16 repetitive regions of interest (ROI) between the trapezium-shaped pillars in the chips. Among the images, grayscale fluorescence images of F-actin staining (red channel) were used for subsequent image analyses. In addition, each ROI was image-captured along the *z*-axis using 27 slices within full thickness. Six microfluidic chips were used for each GRC level, and 2,592 raw images were captured from a single microfluidic chip.

### 2.4 Dataset and preprocessing

The prepared raw image dataset was preprocessed prior to deep learning. First, all images captured from a single 3D microfluidic BC chip with GRC cells were fully stitched and cropped on an ROI-basis to enhance uniformity. Among the 27 slices of *z-*stack, we selected slices with *z* = 1, 3, 5, 14, 23, 25, and 27 for the classification of deep learning applications. Consequently, the total dataset comprised 2,674 GRC images (GRC Level 0: 658, Level 1: 672, Level 2: 672, and Level 3: 672). The shortcomings of the current dataset include the relatively small number of samples and a mild imbalance in the distribution for training data. These issues were addressed using data augmentation method (see results and discussion section for more details).

### 2.5 Deep learning

The architecture of the BC classification depending on the GEM-resistance level was developed based on the CNN (Krizhevsky et al., 2012). The network contained three pairs of convolutional layers, each of which was followed by a max-pooling layer and two fully connected layers. The output of the last fully connected layer is linked to a 4-way softmax layer that returns an array of probability scores to classify the chip images into four classes (i.e., Levels 0, 1, 2, and 3). The rectified linear unit (ReLU) non-linearity $f\left(x\right)=\max\left(0,x\right)$ (Nair and Hinton, 2010) was applied to the output of the convolutional layers and the fully connected layer as activation functions. The first convolutional layer filtered the 64 × 64 × 3 input images with 16 kernels of size 3 × 3 × 3 with a stride of one pixel. The second and third convolutional layers contained 32 and 64 kernels, respectively, with a of size 3 × 3 × 3, respectively. The fully connected layers had 128 and 4 neurons, respectively.

The parameters of the CNN θ were then trained by minimizing the cross-entropy loss function $l\left(\theta \right)$ :

$$\hat{\theta }=\mathrm{argmin}\;l\left(\theta \right)$$

$$l\left(\theta \right)=-\sum_{n=1}^{N}y_{n}\left(x_{n}\right)\log\left(f_{\theta }\left(x_{n}\right)\right),$$ (1)

where *x* represents the input data, *y* represents the corresponding class label, and *f* represents the CNN. We used the Adam optimizer (Kingma and Ba, 2014) with a batch size of 32. The gradient update rules for the parameters in Eq. (1) are as follows:

$$\theta_{t+1}=\theta_{t}-\frac{\alpha }{\sqrt{\hat{v_{t}}+\epsilon }}\hat{m_{t}},$$

$$m_{t}=\beta_{1}m_{t-1}+\left(1-\beta_{1}\right)g_{t},$$

$$v_{t}=\beta_{2}v_{t-1}+\left(1-\beta_{2}\right)g_{t}^{2}$$ (2)

where $\theta_{t}$ denotes the *t*-th update of the parameter θ and $g_{t}=\nabla_{\theta }f_{t}\left(\theta \right)$ is the vector of the partial derivatives of $f_{t}\left(\theta \right)$ with respect to θ . $\beta_{1}$ and $\beta_{2}$ are the hyperparameters that control the exponential decay rates of the moving averages of the gradient ( $m_{t}$ ) and the squared gradient ( $v_{t}$ ), and α denotes learning rate. The default values for $\beta_{1}$ , $\beta_{2}$ , and ϵ were set to 0.9, 0.999, and $10^{-7}$ , respectively.

In this study, we further optimized the learning rate α in Eq. 2 using learning rate schedules of either exponential decay or step decay and compared the classification performance with that based on the default value of 0.001 for α . The model for the exponential learning rate is given as follows:

$$\alpha_{t}=\alpha_{0}e^{-kt},$$ (3)

where $\alpha_{0}$ represents the initial learning rate ( $\alpha_{0}$ = 0.0001 or 0.001), *k* is a hyperparameter (*k* = 0.1), and *t* is an epoch number.

The model for the step decay learning rate is as follows:

$$\alpha_{t}=\alpha_{0}\eta^{-\lfloor t/\gamma \rfloor },$$ (4)

where $\alpha_{0}$ = 0.0001 or 0.001, ⌊x⌋ denotes the floor function of *x*, and hyperparameters η and γ were set to 2.0 and 5.0, respectively. The decay of learning rates was determined using Eq. 3 or Eq. 4, each with different initial values.

### 2.6 Data augmentation

The current dataset lacks a large number of labeled training datasets. To reduce overfitting and improve the classification results in the GRC images, we employed the classic form of data augmentation, which can artificially enlarge the dataset using label-preserving transformations (Krizhevsky et al., 2012). The data augmentation method consisted of randomly translating the images vertically, flipping half of the images vertically, and zooming in on the images.

In an ablation study, we performed a CNN-based classification analysis without utilizing data augmentation methods and predefined learning rate schedules. The network architecture and parameters remained consistent when compared with the other methods used in this study.

### 2.7 Evaluation performance

The performance of the GRC classification was evaluated using a 10-fold cross-validation and a confusion matrix. The dataset was divided into 10 partitions of equal sizes. For each partition, the CNN model was trained on nine partitions and validated on the remaining partitions. The performance measures of the accuracy and confusion matrix were computed on a test dataset. We used Keras and TensorFlow (version 2.5.0) to implement the CNN model for classifying GRC images. CNN training was performed using an NVIDIA 24 GB GeForce RTX 3090 GPU card.

From the confusion matrix, the following metrics were calculated for each GRC level to quantitatively evaluate classification performance:

$$Accuracy=\frac{TP+TN}{\left(TP+TN+FP+FN\right)},$$

$$Precision=\frac{TP}{TP+FP},Recall=\frac{TP}{TP+FN},F1\;score=2∙\frac{Precision∙Recall}{Precision+Recall},$$

$$Sensitivity=\frac{TP}{TP+FN},Specificity=\frac{TN}{TN+FP}$$ (5)

where TP, TN, FP, and FN represent true positive, true negative, false positive, and false negative, respectively. The receiver operating characteristic (ROC) curve was then plotted using the true positive (i.e., sensitivity) and false positive (i.e., 1 – specificity) rates, both of which are based on Eq. 5. Finally, the area under the curve (AUC) was calculated to evaluate model performance.

## 3 Results and discussion

**FIGURE 1:**
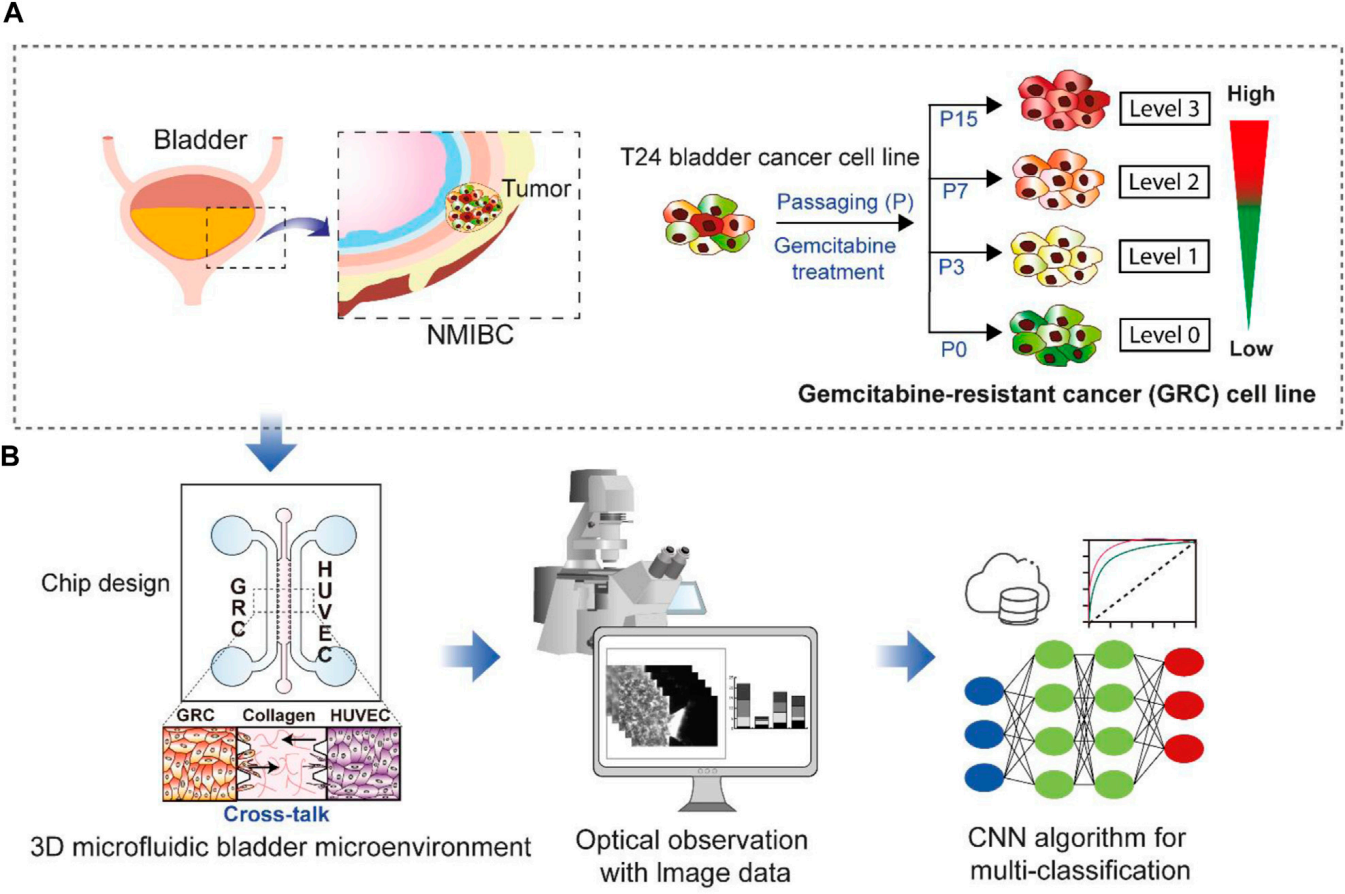
Schematic showing the research purpose and strategies. **(A)** Non-muscle-invasive bladder cancer (NMIBC) is a common type of bladder cancer that exhibits a high rate of resistance to anticancer drugs. The T24 cell line has differential levels (Levels 0, 1, 2, and 3) of gemcitabine resistance established through repetitive subculture (P0, P3, P7, and P15). **(B)** These four different cell lines were used for 3D cell culture in a microfluidic chip to create a bladder cancer microenvironment by co-culturing vessel cells (HUVECs). The resulting images were subsequently subjected to multi-class classification using a convolutional neural network (CNN) to discriminate the cell types based on gemcitabine resistance levels. GRC: Gemcitabine-resistant bladder cancer; HUVEC: Human umbilical vein endothelial cells.

In this study, we explored the potential of classifying previously established GRC cells and predicting GEM resistance in BC using a CNN model. As identified in the previous work (Mun et al., 2022), the GRC cells showed increasingly aggressive phenotypes according to the level of GEM resistance and stepwise changes in the gene expression profile, including 23-gene signatures, revealing that four different GRC cell lines differentially expressed genes associated with specific biological functions. These results contributed to the development of a chemoresistance score based on the 23-gene signature; however, utilizing cellular phenotypes and CNN algorithms could be useful as a more intuitive prediction method for anticancer drug resistance. To achieve this, we utilized a 3D microfluidic chip to create a BC microenvironment depending on different GEM resistance levels by co-culturing with vessel cells, obtained a large amount of image data, and used these data for CNN classification. Figure 1 illustrates the overall scheme and strategy used in this study.

**FIGURE 2:**
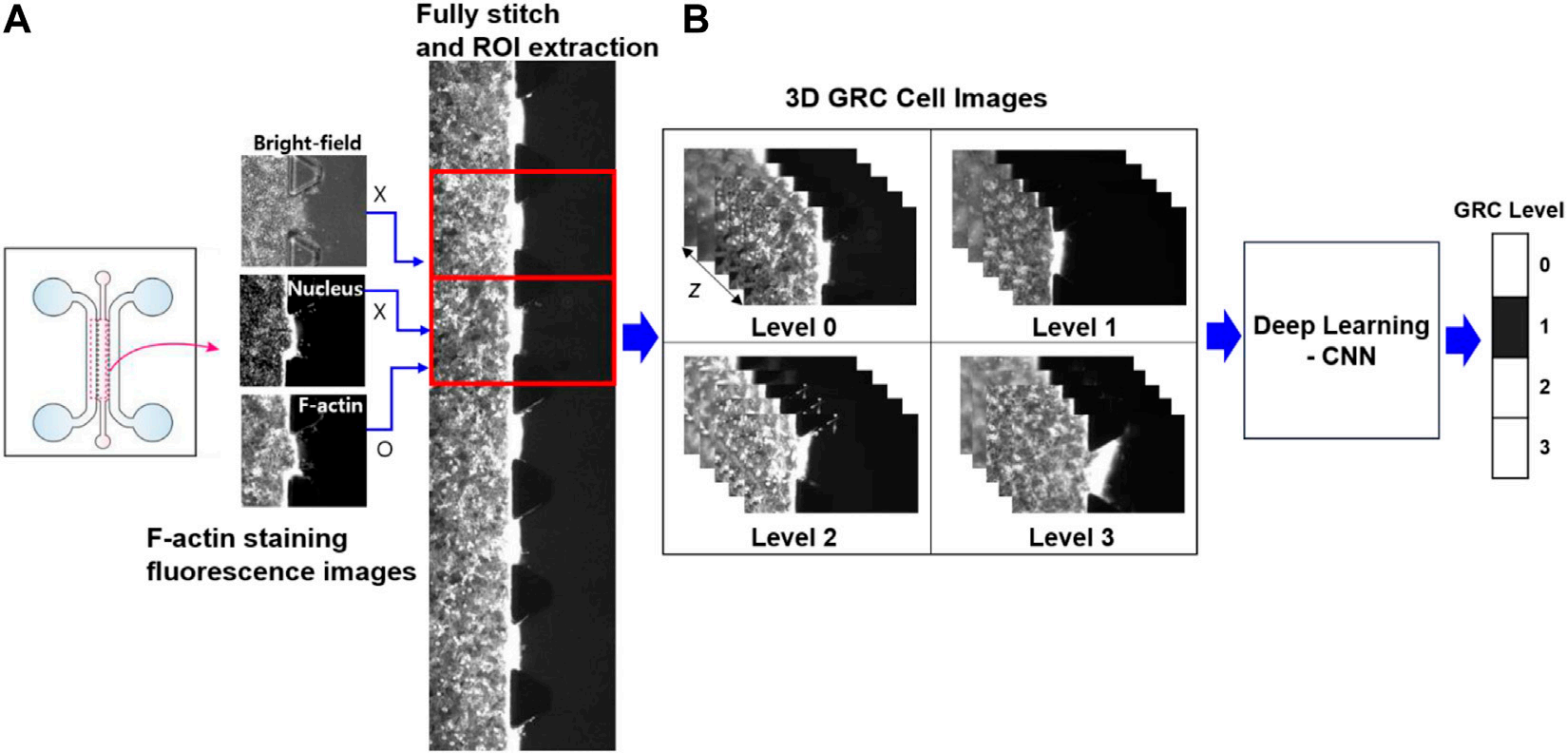
Schematic of image preprocessing and deep learning analysis. **(A)** In fully stitched images acquired from a microfluidic chip, 16 regions of interest (ROI) between the trapezium-shaped pillars were uniformly extracted to represent the cell mobility and the surrounding tissue at the center of each image. **(B)** From a *z*-stack of 27 slices for 3D GRC cell images (F-actin staining; red channel), specific slices (*z* = 1, 3, 5, 14, 23, 25, and 27) were selected for deep learning analysis. This selection prevented redundant features from appearing in adjacent slices along the *z*-axis. Here, Levels 0, 1, 2, and 3 represent gemcitabine resistance levels. GRC: gemcitabine-resistant bladder cancer; CNN: convolutional neural network.

Prior to CNN analysis, a dataset of raw images was further preprocessed to enhance image uniformity. In fully stitched *z*-stack images, 16 repetitive unit-ROI were cut and separated to position the cell and tissue region in the center of the image; appropriate *z*-slices were chosen to enhance the accuracy of classification because the visual variations in nearby slices along the *z*-axis were negligible and may provide duplicate features in deep learning (Figure 2). In our CNN analysis, only grayscale fluorescence images (F-actin staining) were used because they provide morphological features of cells and are more suitable for analysis than bright-field images containing micropillars that the CNNs could potentially perceive as noise.

The images (2,674 images) were randomly shuffled and split into training, validation, and test sets. Specifically, the training, validation and test data were selected as the GRC images originating from different microfluidic chips to ensure that the test sets were disjointed from the training and validation sets. Accordingly, 90% of the data was used as the training set, and the remaining 10% was used as the validation set. The numbers of data for each GEM-resistance level of the GRC cells are summarized in Table 1.

**TABLE 1:** Datasets of 3D microfluidic GRC images for training/validating/testing process.

Dataset	Level 0	Level 1	Level 2	Level 3	Total
Training	492	504	504	504	2,004
Validation	54	56	56	56	222
Test	112	112	112	112	448
Total	658	672	672	672	2,674

Levels 0, 1, 2, and 3 represent gemcitabine resistance levels. GRC, gemcitabine-resistant bladder cancer.

Each dataset (Levels 0, 1, 2, and 3) lacked a sufficient number of labeled training datasets. The distribution of samples across the classes was slightly biased. The class distribution was summarized as percentages of the training dataset: 24.6% in the first class, 25.2% in the second class, 25.2% in the third class, and 25.2% in the fourth class. To the best of our knowledge, there are no strict criteria for defining the degree of data imbalance. However, data imbalance can be categorized as mild to extreme, based on the proportion of the minority class (Google for Developer, 2023: https://developers.google.com/machine-learning/data-prep/construct/sampling-splitting/imbalanced-data). According to this criterion, the current data was mildly imbalanced. Empirically, neural networks can handle mildly imbalanced data (ao Huang et al., 2022). However, to address class imbalance and the challenge of small sample sizes in the training data, we employed a classic form of data augmentation. This approach has been extensively used to reduce overfitting and improve the classification results of these data distributions through an oversampling process (Shorten and Khoshgoftaar, 2019). Specific parameters for data augmentation are shown in Table 2.

**TABLE 2:** Methods and parameters for images augmentation process.

Method	Parameter
Vertical Flip	True
Zoom	$\left[0.1\;0.2\right]$
Vertical Translation	$\left[-0.05\;0.05\right]$

**FIGURE 3:**
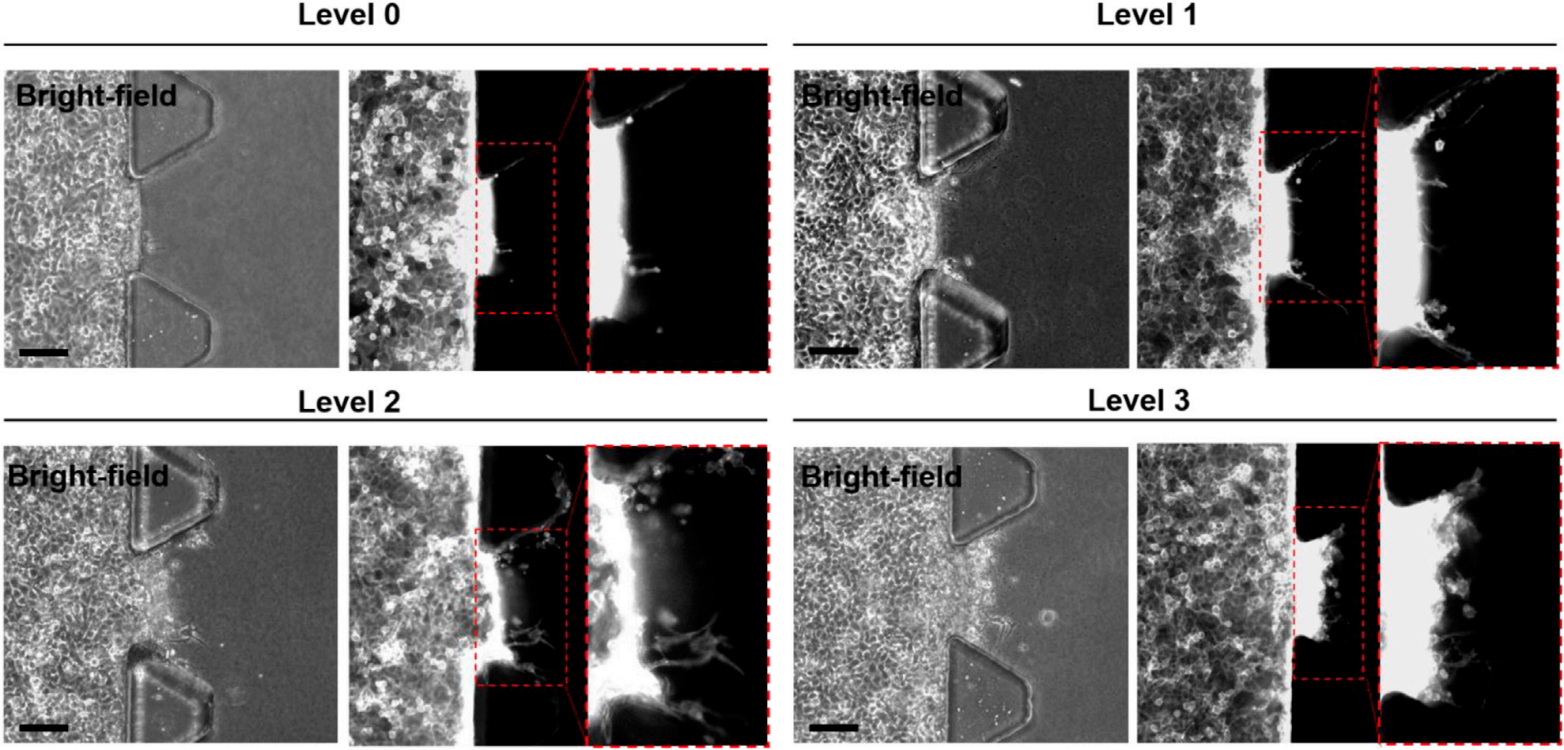
Representative bright-field and fluorescence (F-actin staining; red channel) images of microfluidic GRC models according to the gemcitabine resistance levels (Levels 0, 1, 2, and 3). The inset images are enlarged images of the dotted box region. Scale bars represents 100 µm. GRC: gemcitabine-resistant bladder cancer.

Representative images obtained from each microfluidic GRC model according to the GEM resistance levels are shown in Figure 3. Cells invaded the gel in microfluidic chips depending on their GEM-resistance levels (Mun et al., 2022). As shown in our previous results (Supplementary Figure S1), quantitative image analysis using ImageJ software also showed an increasing tendency with statistically significant differences in the maximum infiltration distance, infiltration area, and number of infiltrating cells, as the cell culture phase increased. In this case, only 15 ROIs were used for each GRC level due to the limitations of manual image analysis. Although statistically significant, the degree of infiltration observed in BC cells during this experiment was not as pronounced as that in other aggressive cancer types, such as lung and brain cancers, as observed in our previous studies (Jeong et al., 2020; Hong et al., 2021). Additionally, the clustered cell morphology, as shown in Figure 3, makes it difficult to quantitatively analyze cells using conventional image analysis tools.

In some studies, the migration speed of cancers has been linked to collagen matrix characteristics such as concentration, composition, and stiffness. These findings indicate that GRC exhibits slow migration in pure collagen with sensitivity to low concentration of collagen (Laforgue et al., 2022) or another hybrid matrix containing Matrigel (Anguiano et al., 2017). These gel conditions should be considered in future studies. In addition, GRC cells infiltrated in response to HUVECs co-cultured in the opposite side channel of the same microfluidic chips, whereas HUVECs that underwent infiltration or angiogenic sprouting were not observed (data not shown). BC cells and HUVECs play interactive roles in the tumor microenvironment (TME) (Huang et al., 2019); however, HUVEC migration was not significant. One report revealed that co-cultured T24 cells exhibit increased proliferation and migration with the support of HUVECs, while co-cultured HUVECs grow slower than mono-cultured cells owing to the changes in cellular energy metabolism in TME (Li et al., 2020). This insight could potentially explain the observed phenomena. Non-obvious changes or quantification using a limited number of samples may result in poor discrimination of cell types and subsequent experimental decisions. Therefore, an advanced classification technique, such as a CNN classification model, is required to accurately analyze cell features with an increased sample size.

**FIGURE 4:**
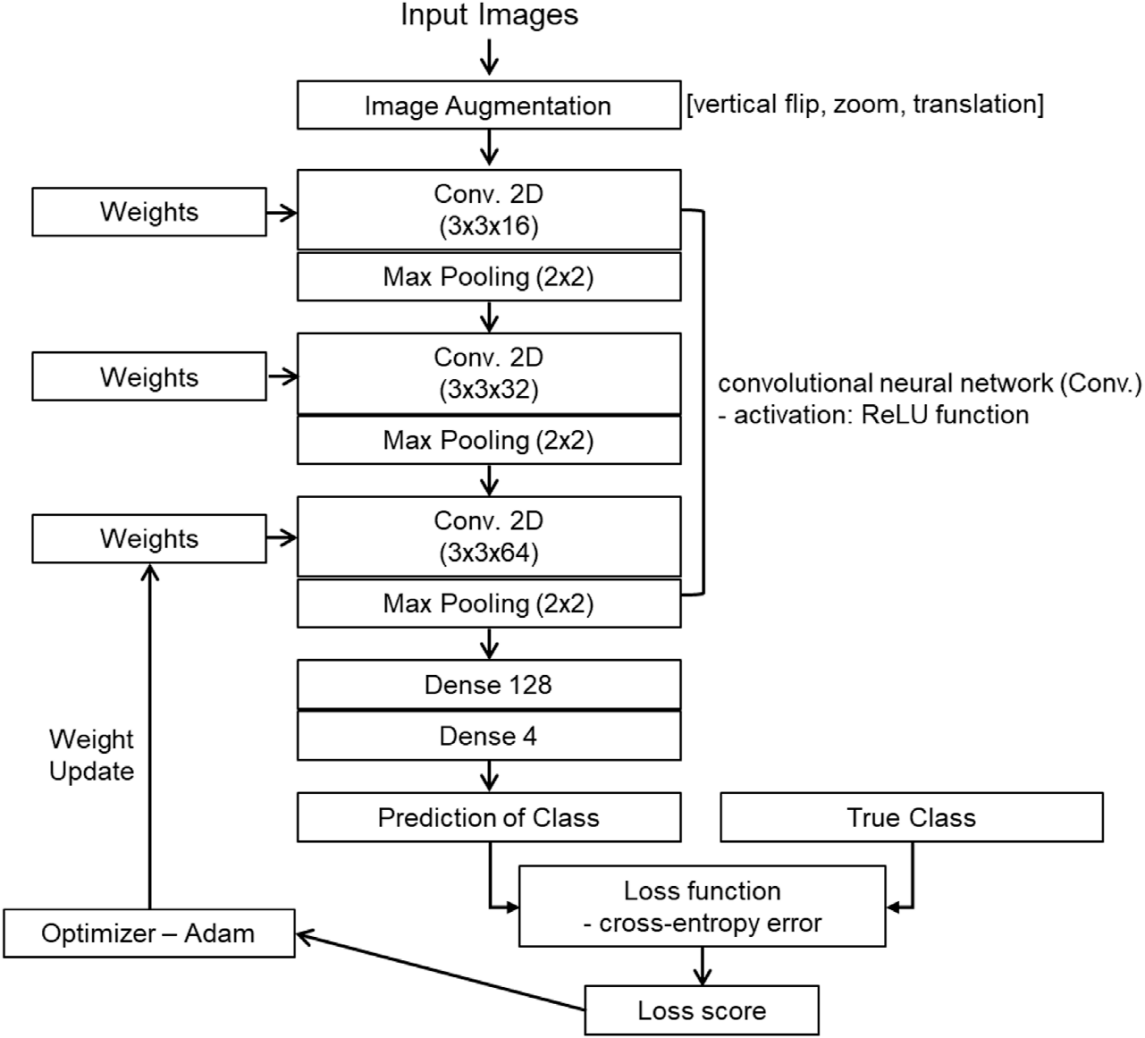
Architecture of the BC classification based on the gemcitabine resistance level. The proposed CNN mainly comprised three sets of convolutional layers and fully connected layers. The network parameters were trained by minimizing the cross-entropy loss function using the Adam optimizer. In this process, we fine-tuned the learning rate using predefined schedules, including exponential or step decay. The trained network was ultimately tested with unseen data to validate its effectiveness in predicting the four levels of gemcitabine resistance from the BC cell images. CNN: convolutional neural network; BC: bladder cancer.

**FIGURE 5:**
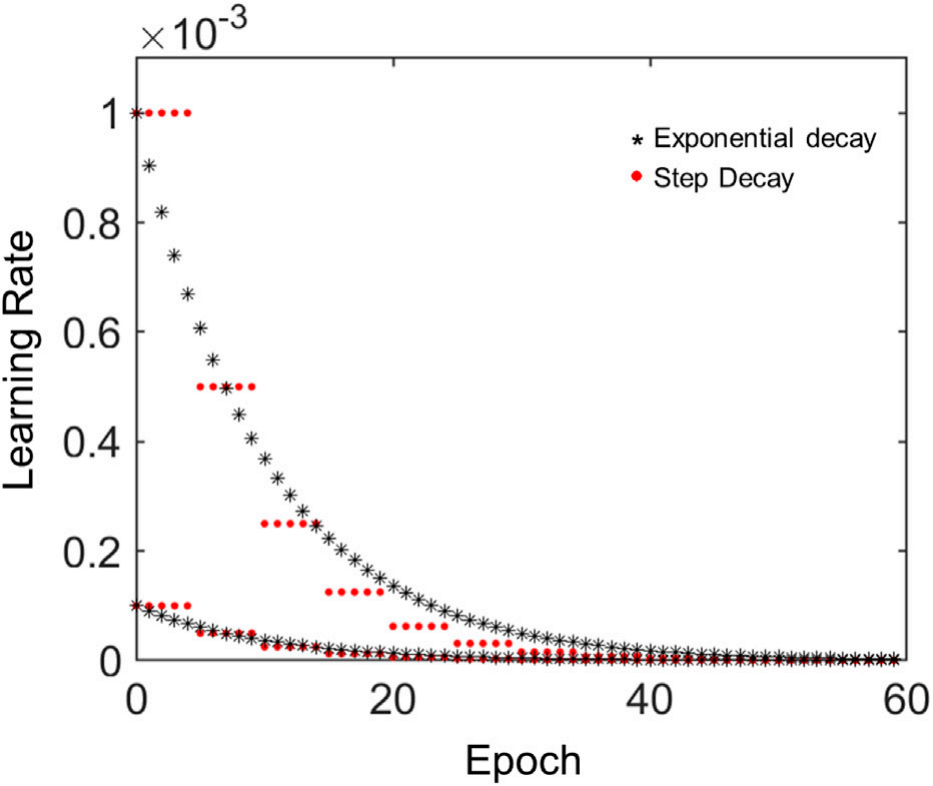
Learning rate changes across epoch numbers according to predefined schedules. The red dots represent the learning rates calculated using the model for step decay. The black asterisks represent the learning rates calculated using the model for exponential decay. In both cases, we tested two models having initial learning rates of 0.0001 or 0.001.

The architecture of the CNN-based GRC classification involves convolution, pooling, and fully connected layers (Supplementary Table S1). An array of probability scores was returned to classify the GRC images into four classes. The process diagram is shown in Figure 4. To evaluate the performance of the CNN multi-class classification model for our microfluidic GRC models, metric scores were calculated. A confusion matrix for each fold of the 10-fold validation was obtained from the trained network. Six metrics, including accuracy, precision, recall, F1 score, sensitivity, and specificity, were then calculated using true positive (TP), true negative (TN), false positive (FP), and false negative (FN), as shown in Eq. 5. Prior to the distribution of accuracy across the GRC levels, we compared the CNN accuracy results for the classification of GRC levels based on data augmentation and predefined learning rate schedules (Figure 5). As shown in Table 3, the CNN without data augmentation and learning rate schedules exhibited the poorest performance in classifying GRC cells. When employing data augmentation, all metric scores of the CNN with learning rate schedules updated at every epoch were consistently higher than those of the CNN with constant learning rates. In particular, using a step decay learning rate with an initial value of 0.001 led to the best performance in the classification of GRC images. The corresponding metric scores, averaged over ten folds and four GRC levels, were as follows: accuracy, 95.2%; precision, 91.2%; recall, 90.5%; F1 score, 90.5%; sensitivity (true positive rate, TPR), 90.5%; and specificity (true negative rate, TNR), 96.8%. Further details regarding the total accuracy calculated from each fold of the training data are provided in Supplementary Table S2.

**TABLE 3:** Performance of the CNN classification for the 3D microfluidic GRC model.

Methods		Accuracy	Precision	Recall	F1 score	Sensitivity	Specificity
Without Aug.	α=0.0001	78.0±2.2	50.3±3.5	55.9±4.3	50.2±4.0	56.0±4.5	85.3±1.5
	α=0.0001	75.1±1.8	46.1±4.4	50.2±3.6	44.3±3.6	50.2±3.8	83.4±1.3
Constant	α=0.0001	91.7±2.9	85.5±5.4	83.4±5.8	83.1±5.9	83.4±6.1	94.5±2.0
	α=0.001	90.3±3.4	85.0±4.4	80.7±6.8	79.5±8.2	80.7±7.1	93.6±2.4
Exp. Decay	$\alpha_{0}=0.0001$	93.5±3.0	88.7±5.4	87.1±6.0	86.6±6.6	87.1±6.3	95.7±2.1
	$\alpha_{0}=0.001$	93.4±1.8	88.0±3.4	86.9±3.6	86.8±3.6	86.9±3.8	95.6±1.3
Step Decay	$\alpha_{0}=0.0001$	93.3±2.6	88.4±4.7	86.6±5.2	86.3±5.5	86.6±5.5	95.5±1.8
	$\alpha_{0}=0.001$	95.2±1.7	91.2±3.1	90.5±3.3	90.5±3.3	90.5±3.5	96.8±1.2

Metric scores were calculated using Eq. 5 and the output of the proposed CNN network with augmentation but different learning schedules. In an ablation study, the performance of a CNN-based classification analysis without utilizing data augmentation methods and predefined learning rate schedules was also evaluated. CNN, convolutional neural network; Aug., augmentation; Exp., exponential; GRC, gemcitabine-resistant bladder cancer.

**FIGURE 6:**
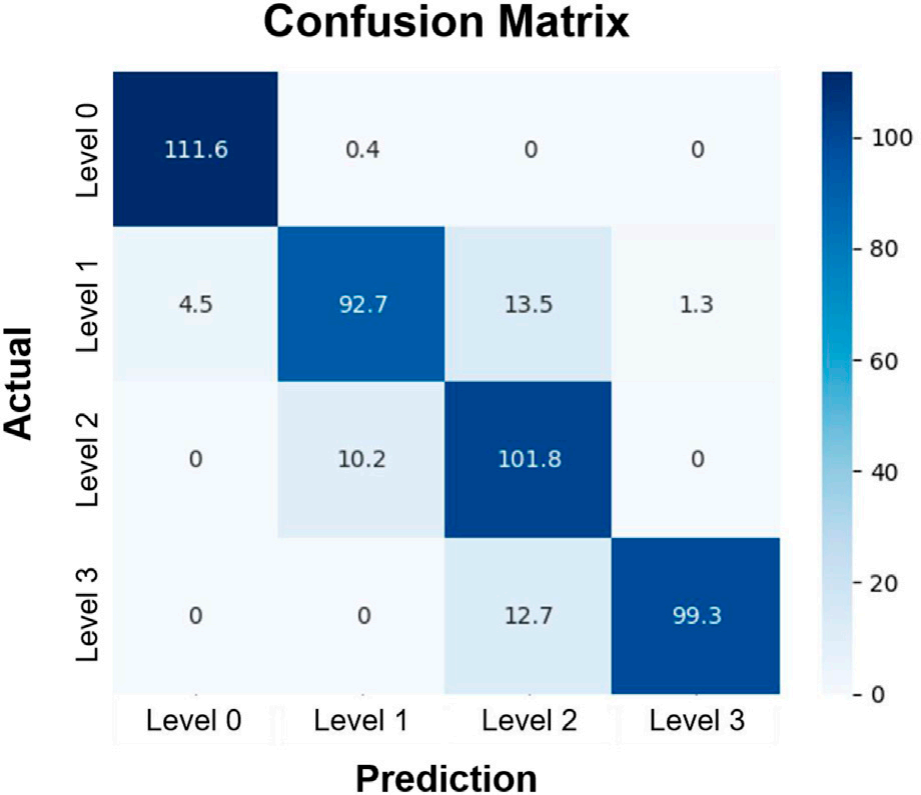
Confusion matrix of the classification of GRC levels. Rows and columns represent the actual and predicted GRC levels of experimental data, respectively. Each element of the matrix indicates the number of data that fell into the specified category, averaged over 10 folds. The test data included 112 images in each GRC level. In this classification, we used the convolutional neural network with the step decay learning rate ( $\alpha_{0}$ = 0.001). GRC: gemcitabine-resistant bladder cancer.

In the remaining analysis, we further assessed the multi-class classification performance of the CNN depending on the GRC levels, using the step decay learning rate ( $\alpha_{0}=0.001$ ). Figure 6 shows the confusion matrix. The rows and columns represent the actual and predicted GEM resistance levels, respectively, from the experimental data. Each element of the matrix indicates the number of data that fell into the specified category, averaged over 10 folds. The CNN trained in this study accurately predicted the GRC level of the image when unseen test data were input into the network. For instance, out of 112 testing data for GRC Level 0, the trained CNN predicted an average of 111.6 samples with the correct GRC Level 0 and 0.4 samples with the incorrect GRC Level 1. Therefore, the ratio of TP to FN was 111.6:0.4. The corresponding sensitivity was TP/(TP + FN) = 111.6/112 = 99.6. The sensitivity values showed a slight decrease across GRC levels 1, 2, and 3: 82.8, 90.9, and 88.6, respectively, when compared with GRC level 0. However, the values of specificity and accuracy consistently remained above 90.0 across all GRC levels. The accuracy, sensitivity, and specificity of the network for each GRC level are summarized in Table 4.

**TABLE 4:** Evaluation of the CNN classification across GRC levels.

	Accuracy	Sensitivity	Specificity
Level 0	98.9±1.0	99.6±1.7	98.7±1.4
Level 1	93.3±2.9	82.8±8.8	96.8±1.6
Level 2	91.9±2.9	90.9±5.0	92.2±2.9
Level 3	96.9±1.7	88.6±6.2	99.6±0.7

Metric scores were calculated using Eq. 5 and a trained network with step decay learning rates. Each value represents the mean and standard deviation of metric scores over 10 folds. Levels 0, 1, 2, and 3 represent gemcitabine resistance levels. CNN, convolutional neural network; GRC, gemcitabine-resistant bladder cancer.

**FIGURE 7:**
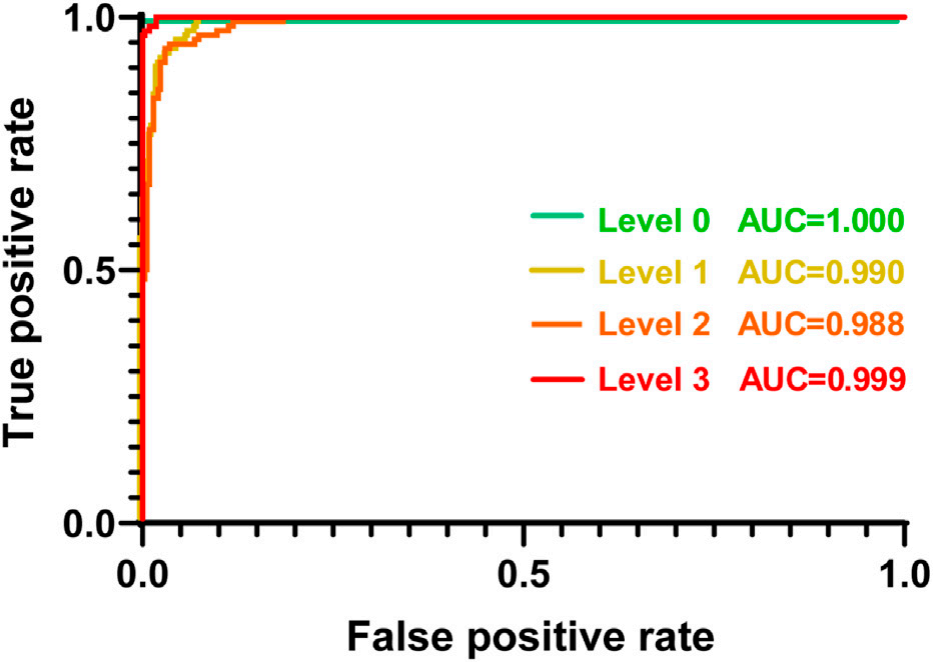
Receiver operating characteristic curve (ROC) plot of each GRC level. Each GRC level was discriminated from other levels. AUC: area under the curve; GRC: gemcitabine-resistant bladder cancer.

The ratio of the true positive rate (TPR) to the false positive rate (FPR) based on the proposed CNN network was finally assessed using an ROC curve. As shown in Figure 7, the TPR was markedly higher than the FPR across the entire range of possible decision thresholds. These results were consistently obtained from the four datasets of the GRC levels, although the ratios of TPR to FPR estimated from the datasets of Levels 1 and 2 were slightly lower than those of Levels 0 and 3. We quantified the overall accuracy of the test (i.e., the ratio of TPR to FPR) using AUC. The AUC values of the tests for predicting each level were 1.00, 0.99, 0.98, and 0.99, respectively. These results suggest that the proposed CNN framework can be considered to have an acceptable discriminating ability (Mandrekar, 2010) for the various GRC levels of BC cells. Taken together, it is confirmed that our developed CNN can not only classify the different GRC levels with high accuracy, but also predict the GRC level from randomly provided data with high sensitivity and selectivity.

GEM resistance is a major issue in BC chemoresistance, and its modulating genes and pathways may vary among the sequential GRC cells. For example, our previous study (Mun et al., 2022) indicated that a high level of GRC cells was associated with poor prognosis and a low response rate to other chemotherapeutic drugs such as cisplatin, carboplatin, and doxorubicin. Alternative therapies should be considered in such cases. Additionally, it provided informative results showing that a high level of GRC consistently increased epithelial–mesenchymal transition (EMT)-related gene expression; however, it downregulated the cytosolic DNA and endoplasmic reticulum (ER) stress genes. These different gene patterns based on specific drug resistance levels may provide valuable insights into the selection of adjunctive drug agents (Jia and Xie, 2015; Wu et al., 2021; Liu et al., 2022).

At the current stage of our research, AI classification of GRC levels does not provide a precise criterion for which the level of drug resistance affects drug effectiveness, or which alternative drugs should be considered at each drug resistance level. To date, most studies have addressed either genetics or image-based analysis, and only a few studies have integrated both approaches (Couture et al., 2018; Wulczyn et al., 2020; Ash et al., 2021; Chen et al., 2022; Schneider et al., 2022; Fremond et al., 2023). These combined therapeutic strategies with the integration of genetics hold prognostic potential, providing reference basis for drug choices.

In this study, we applied a CNN-based deep learning method to images obtained from a 3D cell culture platform to classify 3D GRC cell images into four classes based on GEM resistance levels. Conventionally, mono-culture or microfluidic 2D cells have been used for CNN analysis (Zhang et al., 2018; Hashemzadeh et al., 2021; Pérez-Aliacar et al., 2021). However, the 3D microfluidic BC model under cultivation with other types of cells can contribute to better classification efficiency because this platform could provide more appropriate phenotypes of invasive cancer when compared to mono-culture or 2D culture. Previously, CNN was applied to relatively obvious problems in microfluidic chip applications, such as classifying cancer into benign and malignant (Wang et al., 2019) or discriminating between different types of cell lines of different origins (Hashemzadeh et al., 2021). However, multi-class classification (Heenaye-Mamode Khan et al., 2021), such as distinguishing between four different cell lines derived from the same parental cell by morphological features, is much more challenging.

The proposed CNN architecture consisted of three convolutional layers with data augmentation and a step decay learning rate optimized for our dataset. Compared with the conventional method (Mun et al., 2022), deep learning-based analysis automatically extracts discriminative features from data during the training process. The trained network then allowed for predicting the class level of the GRC images with an overall accuracy of 95.2%. The AUC values across the classes confirm the reliable discriminating ability of the proposed network.

Deep learning is a method that enables automatic estimation of representations required for classification or detection from raw data (LeCun et al., 2015). Specifically, deep learning involves multiple successive layers of representation using nonlinear modules. Layered representations can then be learned from the training data using a backpropagation procedure. These deep learning methods have been successfully applied to multiple areas of biomedical imaging, such as image classification, segmentation, recognition, and diagnosis (Shen et al., 2017). However, in the field of organ-on-a-chip technology, only a few recent studies have used deep learning in their research domain (Li et al., 2022; Choi et al., 2023).

CNN (LeCun et al., 1989; Gu et al., 2018), a subtype of deep learning, offers the advantage of reducing the number of parameters through the use of a shared-weight convolutional architecture, while also leveraging image spatial structures via filters. In this study, CNN methodology was applied to 3D cell images acquired from an organ-on-a-chip system. In particular, we utilized a series of image slices acquired at multiple locations within the sample along the optical *z-*axis for the CNN analysis. This approach enabled the utilization of spatial variances within GRC images, both within individual slices and across multiple slices, to classify distinct GRC levels of BC cells.

There are limitations to this study that need to be addressed. One limitation of our study lies in the fact that we did not address the out-of-focus issue, which resulted in blurry images from the acquisition below and above the optimal focal plane. This out-of-focus artifact can be corrected using deblurring algorithms based on a cycle generative adversarial network (GAN) (Zhang et al., 2022). CycleGAN can be used to learn the deblurring filter using a pair of in-focus and the corresponding out-of-focus images. In addition, we used only fluorescence images in our CNN analysis. However, cell staining is time-consuming and may introduce artifacts to cell morphologies during the process. One study reported the potential application of a line detector-based Hough transform (Parlato et al., 2017) for eliminating unnecessary background, barriers, or channels from the images of the microfluidic chip. Applying this method could enable the use of unstained bright-field images for straightforward morphological analysis. These techniques will be explored in future studies.

Finally, the current dataset used in this study consisted of a relatively small number of samples for each class. It is known that overfitting can occur when using a small number of data in training deep neural networks, and this prevents the generalization of trained networks to new testing data (Chollet, 2021). Therefore, in order to overcome this challenge, we further optimized the CNN hyperparameters using standard data augmentation and the predefined learning rate schedules. This resulted in an acceptable classification accuracy of 95.2%, despite the limited number of labeled training datasets. In addition, we attempted to address the limitations of a small dataset by using transfer learning from pretrained networks, MobileNetV2 (Howard et al., 2017) and Xception (Chollet, 2017). Specifically, we tested a pretrained network on ImageNet as a feature extractor and fine-tuned it to fit our dataset (GRC cell images). Supplementary Table S3 shows the accuracy, sensitivity, and specificity of the pretrained networks in the classification of GRC cell images based on GEM resistance levels. Each value represents the mean and standard deviation of metric scores over 10 folds. In the case of our dataset, the performance of transfer learning from a pretrained network was worse than that of training the proposed CNN network from scratch. This could be attributed to the dissimilarity between our dataset, which comprises BC cell images cultured in a 3D microfluidic chip, and the source dataset of the pretrained network, ImageNet. To address this concern, we performed fine-tuning on the pretrained network by unfreezing the high-level layers. This allowed us to retain generic features while enabling the learning of data-specific features from our dataset. However, the highest classification accuracies for MobileNetV2 and Xception were 85.7 ± 1.9 and 79.1 ± 1.0, respectively, both lower than that of the CNN used in this study. Although additional optimization is necessary for the pretrained networks, given the limited size of our unique dataset (i.e., organ-on-a-chip), utilizing a CNN with a small number of layers, augmentation, a step decay learning rate, and training the network from scratch prove to be a suitable approach. Data augmentation still has limitations in generating synthetic images with realistic and natural shapes based on the training dataset because little additional information can be obtained from the conventional modification to the images. Therefore, in future studies, we will incorporate the GAN-based generation of synthetic images for data augmentation (Frid-Adar et al., 2018) into the current CNN framework. This may lead to a better classification accuracy and utilization of 3D CNN for accommodating the 3D structures of BC images along the optical axis.

## 4 Conclusion

In this study, we propose a promising prognostic system that enables the prediction of the extent of anticancer drug resistance in BC by synergistically integrating organ-on-a-chip and deep learning techniques. A large amount of data was acquired from microfluidic chips mimicking the BC microenvironment, according to the different anticancer drug resistance levels, and was processed and analyzed using the CNN algorithm. Given the complex and multiple classifications of GRC cell lines representing the four different characteristics of drug resistance, this integrated system exhibited high performance and accuracy in multi-class classification to predict the level of anticancer drug resistance. The lowest sensitivity and specificity were 82.8%, and 92.2%, respectively. In the future, the application of patient-derived cells to this system is expected to become a feasible method for screening or predicting anticancer drug resistance levels in patients from real culture images. Moreover, the chip systems coupled with an automatic imaging system could accelerate the imaging and overall processing time and can eventually be extended to diagnose the other subtypes of cancers and diseases to make prognostic predictions.

## Data Availability

The original contributions presented in the study are included in the article/Supplementary Material, further inquiries can be directed to the corresponding author.
