# Supplementary material for: Prediction of anticancer drug resistance using a 3D microfluidic bladder cancer model combined with convolutional neural network-based image analysis
Source: Front Bioeng Biotechnol. 2024 Jan 10;11:1302983. doi: 10.3389/fbioe.2023.1302983 (PMC10806080; doi:10.3389/fbioe.2023.1302983)
Supplement: Supplementary file 1 [file DataSheet1.docx]

Supplementary Material

**Prediction of anticancer drug resistance using a 3D microfluidic bladder cancer model combined with convolutional neural network-based image analysis**

**Sungho Tak^1,2^, Gyeongjin Han^1^, Sun-Hee Leem^3^, Sang-Yeop Lee^1^, Kyurim Paek^4^, Jeong Ah Kim^4,5,6*^**

^1^Research Center for Bioconvergence Analysis, Korea Basic Science Institute, Cheongju 28119, Republic of Korea

^2^Graduate School of Analytical Science and Technology, Chungnam National University, Daejeon 34134, Republic of Korea

^3^Department of Biomedical Sciences, Dong-A University, Busan 49315, Republic of Korea

^4^Center for Scientific Instrumentation, Korea Basic Science Institute, Daejeon 34133, Republic of Korea

^5^Department of Bio-Analytical Science, University of Science and Technology, Daejeon 34113, Republic of Korea

^6^Chung-Ang University Hospital, Chung-Ang University College of Medicine, Seoul 06973, Republic of Korea

***Correspondence:**

Jeong Ah Kim

[jakim98@kbsi.re.kr](mailto:jakim98@kbsi.re.kr)

**
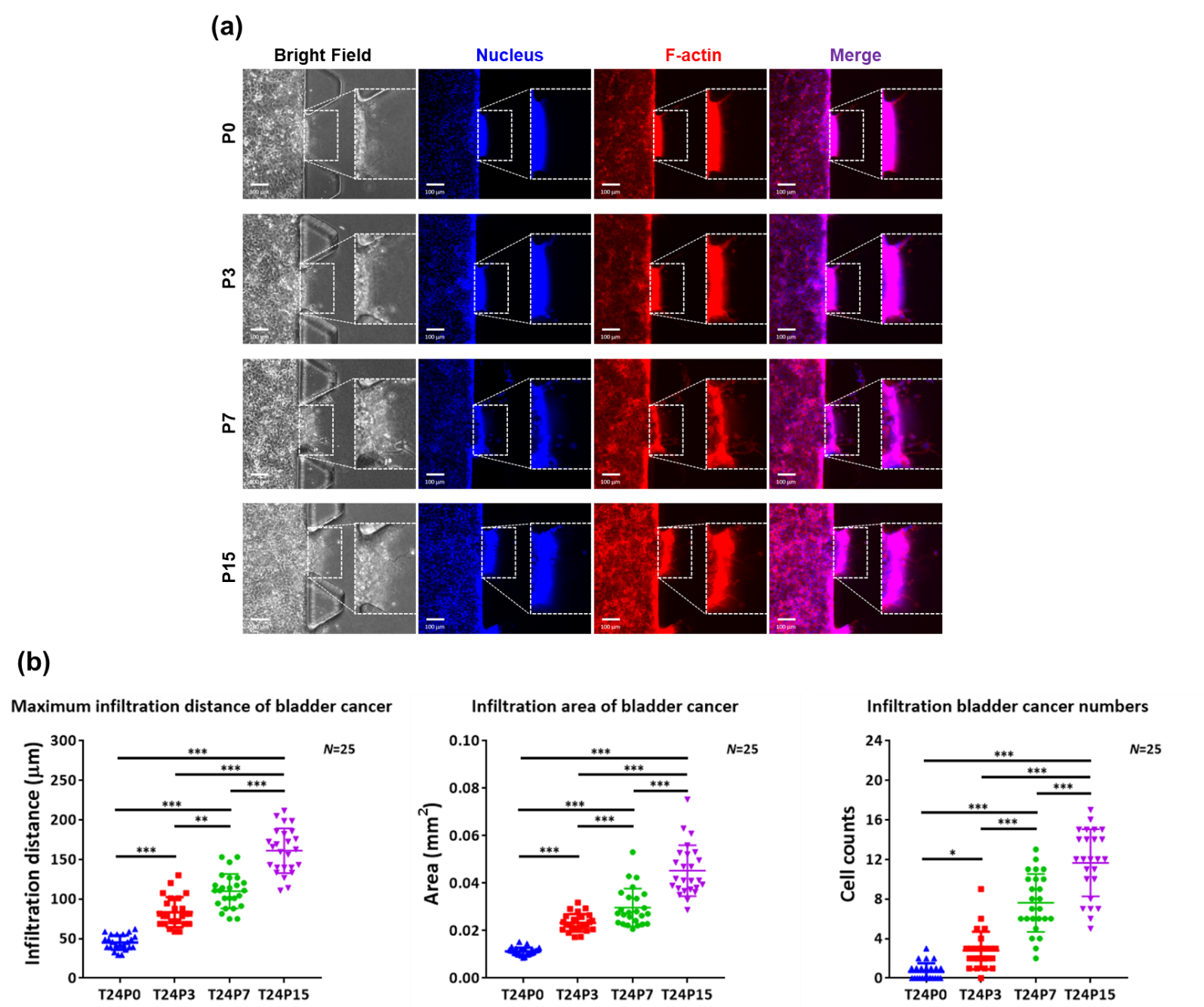
**

**Figure S1. Representative images of the 3D microfluidic GRC cells.** (a) Cells invaded the gel, and the morphological features, including migration pattern, cell shape, and the extent of invasion, exhibited variations depending on the levels of GEM resistance. Scale bar represents 100 µm. (b) Quantitative image analysis of the infiltration distance, infiltration area, and infiltration numbers of the GRC cells using Image J software (data obtained from our group’s previous study; Mun et al., 2022). In the image, phases P0, P3, P7, and P15 correspond to levels 0, 1, 2, and 3, respectively. *N* indicates number of ROI, * *p* < 0.05, ** *p* < 0.01, *** *p* < 0.001. Figure reproduced with permission from the publisher. Copyright 2022 Springer Nature. GRC: gemcitabine-resistant bladder cancer; GEM: gemcitabine.

**Supplementary tables:**

**Table S1. Summary of the CNN model used in this study**

| **Layer (type)** | **Output Shape** | **Parameter #** |
| --- | --- | --- |
| conv2d_1 (Conv2D) | (None, 64, 64, 16) | 448 |
| max_pooling2d_1 (MaxPooling2D) | (None, 32, 32, 16) | 0 |
| conv2d_2 (Conv2D) | (None, 32, 32, 32) | 4,640 |
| max_pooling2d_2 (MaxPooling2D) | (None, 16, 16, 32) | 0 |
| conv2d_3 (Conv2D) | (None, 16, 16, 64) | 18,496 |
| max_pooling2d_3 (MaxPooling2D) | (None, 8, 8, 64) | 0 |
| flatten_1 (Flatten) | (None, 4,096) | 0 |
| dense_1 (Dense) | (None, 128) | 524,416 |
| dense_2 (Dense) | (None, 4) | 516 |
| Total parameters: 548,516  Trainable parameters: 548,516  Non-trainable parameters: 0 | | |

**Table S2. Classification accuracy from the 10-fold cross-validation with different learning rate schedules**

|  | Without Aug. | | Constant | | | Exponential Decay | | | Step Decay | | |
| --- | --- | --- | --- | --- | --- | --- | --- | --- | --- | --- | --- |
|  | 0.0001 | 0.01 | | 0.0001 | 0.001 | | 0.0001 | 0.001 | | 0.0001 | 0.001 |
| Fold 1 | 78.3 | 74.8 | | 93.7 | 86.9 | | 98.1 | 93.8 | | 94.5 | 95.4 |
| Fold 2 | 77.8 | 78.3 | | 86.0 | 86.8 | | 88.4 | 95.3 | | 88.3 | 96.5 |
| Fold 3 | 76.1 | 73.2 | | 92.6 | 94.0 | | 95.8 | 89.6 | | 91.7 | 95.4 |
| Fold 4 | 77.8 | 76.0 | | 90.2 | 93.9 | | 92.7 | 95.4 | | 93.6 | 97.9 |
| Fold 5 | 79.7 | 75.2 | | 95.1 | 91.7 | | 93.4 | 92.0 | | 91.3 | 92.6 |
| Fold 6 | 75.9 | 72.9 | | 89.8 | 88.5 | | 91.1 | 94.6 | | 94.6 | 94.0 |
| Fold 7 | 80.8 | 75.6 | | 92.3 | 87.9 | | 95.4 | 91.5 | | 96.5 | 95.2 |
| Fold 8 | 73.4 | 74.0 | | 93.1 | 85.5 | | 89.3 | 93.1 | | 93.9 | 93.5 |
| Fold 9 | 78.2 | 77.8 | | 95.6 | 95.5 | | 95.3 | 94.8 | | 97.4 | 94.1 |
| Fold 10 | 81.1 | 73.3 | | 88.3 | 92.5 | | 96.0 | 94.5 | | 91.1 | 97.8 |
| Mean | 78.0 | 75.1 | | 91.7 | 90.3 | | 93.5 | 93.4 | | 93.3 | 95.2 |
| Std. | 2.2 | 1.8 | | 2.9 | 3.4 | | 3.0 | 1.8 | | 2.6 | 1.7 |

**Table S3. Performance of transfer learning from a predefined network in classification of GRC cell images based on GEM resistance levels**

| Method | | Accuracy | Sensitivity | Specificity |
| --- | --- | --- | --- | --- |
| MobileNetV2 | Feature Extractor | $73.3\pm2.2$ | $46.7\pm4.5$ | $82.2\pm1.5$ |
|  | Fine-Tuning | $85.7\pm1.9$ | $71.4\pm3.7$ | $90.5\pm1.2$ |
| Xception | Feature Extractor | $75.8\pm1.4$ | $51.7\pm2.8$ | $83.9\pm1.0$ |
|  | Fine-Tuning | $79.1\pm1.0$ | $58.2\pm2.1$ | $86.1\pm1.0$ |
